# Bioactive cationic peptides as potential agents for breast cancer treatment

**DOI:** 10.1042/BSR20211218C

**Published:** 2021-12-07

**Authors:** Marcela Manrique-Moreno, Gloria A. Santa-González, Vanessa Gallego

**Affiliations:** 1 Chemistry Institute, Faculty of Exact and Natural Sciences, University of Antioquia, A.A. 1226, Medellin, Antioquia; 2 Biomedical Innovation and Research Group, Faculty of Applied and Exact Sciences, Instituto Tecnólogico Metropolitano, A.A. 54959, Medellin, Colombia

**Keywords:** antimicrobial peptides, breast cancers, cationic peptides, drug development, new cancer treatments

## Abstract

Breast cancer continues to affect millions of women worldwide, and the number of new cases dramatically increases every year. The physiological causes behind the disease are still not fully understood. One in every 100 cases can occur in men, and although the frequency is lower than among women, men tend to have a worse prognosis of the disease. Various therapeutic alternatives to combat the disease are available. These depend on the type and progress of the disease, and include chemotherapy, radiotherapy, surgery, and cancer immunotherapy. However, there are several well-reported side effects of these treatments that have a significant impact on life quality, and patients either relapse or are refractory to treatment. This makes it necessary to develop new therapeutic strategies. One promising initiative are bioactive peptides, which have emerged in recent years as a family of compounds with an enormous number of clinical applications due to their broad spectrum of activity. They are widely distributed in several organisms as part of their immune system. The antitumoral activity of these peptides lies in a nonspecific mechanism of action associated with their interaction with cancer cell membranes, inducing, through several routes, bilayer destabilization and cell death. This review provides an overview of the literature on the evaluation of cationic peptides as potential agents against breast cancer under different study phases. First, physicochemical characteristics such as the primary structure and charge are presented. Secondly, information about dosage, the experimental model used, and the mechanism of action proposed for the peptides are discussed.

## Introduction: the need for new therapeutic options for breast cancer

Cancer is defined as a broad group of diseases characterized by uncontrolled and abnormal cell growth, which frequently invades adjacent organs or tissues and spreads into the body. The latter feature is known as metastasis and is a principal cause of death from this malignancy. Cancer is the first or second leading cause of death before the age of 70 years in 112 of 183 countries [1]. Breast cancer is the world’s most commonly diagnosed malignancy, according to statistics released by the International Agency for Research on Cancer (IARC) in December 2020 [2]. It can occur in women of any age, including cases with no identifiable cancer risk factors. According to statistics from the World Health Organization (WHO), more than 2.3 million women were diagnosed with breast cancer in 2020, while there were 685000 deaths globally [3]. Male breast cancer is considered a rare disease, accounting for approx. 1% of all breast cancer cases, but, like female breast cancer, its incidence has increased over the past 25 years [4]. The breast comprises glands, including the breast lobes and breast ducts, whose function is to produce milk during the lactation period. The lobes are connected by the mammary ducts, which carry milk to the nipple. The glands and ducts of the breast are embedded in adipose tissue and connective tissue, which, together with lymphatic tissue, form the breast. The pectoral muscle, located between the ribs and the breast, acts as a retaining wall. Finally, the skin covers and protects the entire breast structure [5].

Breast cancer can be classified as carcinoma or sarcoma, depending on which cells become cancerous. Carcinomas are a type of breast cancer that involves the glandular epithelium, and sarcomas arise from the stromal components of the breast, including myofibroblasts and blood vessel cells, in addition, these cancers are rare and uncommon (<1% of the cases). However, in some cases, a breast tumor can be a combination of different cell types [6]. Carcinomas are the most common types of breast cancer that can be identified according to their invasiveness relative to the site of the primary tumor. The two most common types are infiltrating ductal carcinoma, where cancer cells multiply outside the ducts and invade other parts of the breast tissue, and infiltrating lobular carcinoma, in which cancer cells spread from the lobules to nearby tissues [7]. Ductal carcinoma is the most frequent breast cancer (50–75% of patients), followed by invasive lobular carcinoma (5–15% of patients) [8,9]. At the early stage of the pathology, the malignant cells are confined to the duct, do not cause symptoms, and have minimal metastasis potential. The physiological causes behind breast cancer are complex and not entirely understood. Breast cancer is a heterogeneous disease comprising multiple entities associated with distinctive histological and biological features [10], including hormone receptor status and expression [11], clinical presentations and behaviors, and responses to therapy [12–14]. However, certain factors increase the risk of the disease, including family and reproductive history, prolonged consumption of oral contraceptives, harmful use of alcohol and tobacco, increasing age, obesity, radiation exposure, and postmenopausal hormone therapy. Meanwhile, physical activity is considered protective [15–17]. The most frequent symptoms of breast cancer are a breast lump, change in nipple appearance; alteration in size, shape, or appearance of a breast; and redness or other alterations in the skin surrounding the nipple (areola).

The WHO Global Breast Cancer Initiative (GBCI) prevention and detection programs have succeeded in reducing breast cancer mortality; achieving an annual breast cancer mortality reduction of 2–4% per year, representing thousands of lives saved [18]. Unfortunately, epidemiological data project that the number of new cases will persistently increase over the next two decades. According to the IARC, between 2020 and 2040, 3.2 million women will be diagnosed with breast cancer, and almost 1 million will die [19]. The probable outcomes of patients depend on the country and the strength of the health system, third-world countries having poorer outcomes in respect of early detection, treatment quality, and survivorship care [20–24]. Considering this, health programs aimed at improving the detection of signs and symptoms of early breast cancer, so that patients are referred to diagnostic services in the first stages of the disease, are essential in order to reduce the number of cases [25]. Breast cancer treatments can be highly effective when the disease is identified early. However, as the disease progresses, malignant cells invade the surrounding tissues, lymph nodes, and multiple organs in the body, including the lungs, liver, brain, and bones. Once metastasis has occurred, the possibility of patient survival is reduced [26–28]. Therefore, early detection of breast cancer is vital for the management and prediction of breast cancer evolution.

Breast cancer treatments can be local or systemic, treatment selection depends on several factors. During the diagnostic process, it is essential to determine the characteristics of the tumor and the number of affected nodes to avoid recurrence of the disease [29]. In the past, radical mastectomy was traditionally the treatment for early-detected cases of invasive breast cancer. This allowed local control of the disease, since the goal of this treatment was to remove the affected area, avoiding metastasis. However, breast-conserving surgery (also called a lumpectomy, quadrantectomy, or partial mastectomy) is considered a less aggressive option, prioritizing the preservation of healthy breast tissue that is not affected by the disease [30]. Although axillary lymph nodes are usually compromised in breast cancer, their evaluation provides valuable information about the stage and prognosis of the disease. In the sentinel node biopsy (SNB), a dye or a radioactive tracer is used to detect the lymph nodes under the arm involved in the spread of cancer from the breast. This procedure involves the removal of one or several lymph nodes, lowering the risk from the surgery, lymphedema and side effects like pain, numbness, swelling, and decreased mobility of the affected arm [31].

Radiation therapy is based on the use of high-energy ionizing radiation to destroy cancer cells and reduce the tumor size. There are two primary forms of radiotherapy: external beam radiation, which is directed at the outside of the body, and internal radiation, also referred to as brachytherapy, in which the radioactive source is delivered inside the body for a short period [32,33]. Radiotherapy plays a significant role in treating breast cancer. It can be used as a sole treatment in order to permanently eradicate the primary tumor and regional node metastasis, or in combination with surgery, in both cases preoperatively. It can also be used to inactivate a large proportion of clonogenic tumor cells and shrink inoperable or borderline operable tumors. Finally, it can be used postoperatively, to eliminate residual subclinical cancer deposits on the tumor bed or positive margins remaining in the tissues surrounding the resected area. However, radiation therapy in metastatic disease is almost entirely reserved for the palliation of symptoms [34].

Modern approaches have incorporated new techniques based on improved understanding of breast cancer, in order to optimize and individualize breast cancer treatment. Gene expression techniques have made it possible to differentiate types of intrinsic breast cancer genes, which has changed approaches to the disease from being based on tumor burden to a focus on specific biological characteristics [6,35]. The main differences that breast cancer cells express and define in the treatment are the human epidermal growth factor receptor 2 (HER2-positive), hormone receptor-positive breast cancer, BRCA gene mutations, and triple-negative breast cancer (TNBC) [36]. Trastuzumab, pertuzumab, and margetuximab are monoclonal antibodies that bind to the HER2 protein on cancer cells, preventing the cells from growing. Therapy with HER2-targeted treatments combined with chemotherapy, has led to an improvement in the clinical outcomes of patients [37]. Targeted therapy for hormone receptor-positive breast cancer includes palbociclib, ribociclib, and abemaciclib, which block CDK4 and CDK6. In hormone receptor-positive breast cancer cells, blocking these proteins helps stop proliferation of the cells; which can delay the progression of cancer [38]. Although different types of medication are available, they have a different mechanism of action to chemotherapy drugs and frequently have side effects. Common targets in breast cancer include olaparib, talazoparib and PARP inhibitors, which have been studied in women with breast or ovarian cancers associated with deleterious germline mutations in BRCA1 and BRCA2. In terms of median progression-free survival, they have proven efficacy [39]. The cancer cells in TNBC lack estrogen and progesterone receptors and overproduce the HER2 protein. Some drugs, such as pembrolizumab and iniparib, are currently in clinical trials with promising effects in TNBC, but serious adverse events have been reported [40]. Although therapies directed at these receptors are administered to decrease their activity, there are limitations related to adverse effects. For example, in the case of endocrine therapy, significant side effects are menopause and arthropathy; while less common but potentially fatal side effects are are pulmonary embolism, endometrial cancer, and osteoporotic fracture [41]. The main limitations of monoclonal antibodies are their size and high molecular weight, which are related to their tissue penetration properties. This hinders their internalization into solid tumors [34]. Furthermore, nonspecific uptake of these molecules has been reported in parts of the endothelial reticulum system such as the liver, spleen, and bone marrow [42,43].

The complexity of cancer and the burden it represents for the health system necessitates the intervention of multiple areas of science focused on the search for new breast cancer treatment strategies. Current therapeutic options involve long treatments with numerous side effects that affect the quality of life of patients. Therefore, the search for new antiproliferative agents continues to be a priority. These compounds must be capable of eliminating cancer cells and be selective enough not to cause damage to the healthy cells of the tissue surrounding the lesions. Therefore, it is necessary to develop new therapeutic strategies based on systems that increase selectivity for use individually or synergistically with conventional breast cancer procedures. These can offer patients more selective and less cytotoxic alternatives, thus improving their quality of life.

## Cationic peptides as agents against breast cancer

**Figure 1:**
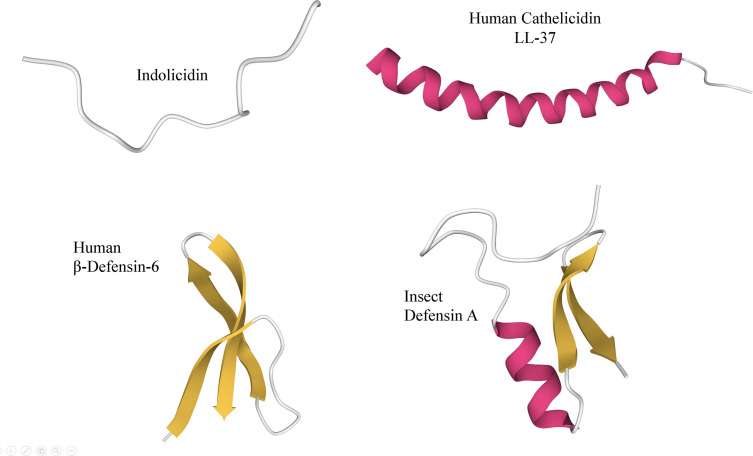
Structural diagram of representative BCPs generated using PyMOL Indolicidin (Protein Data Bank ID: IG89), Human Cathelicidin LL-37 (Protein Data Bank ID: 2K6O), Human β-Defensin-6 (Protein Data Bank ID: 1ZMQ), and Insect Defensin A (Protein Data Bank ID: 1ICA). The colors represent the secondary structures.

Several studies have shown that cancer cells develop multidrug resistance to chemotherapeutics [44–47]. Changes are induced at the cellular level that include overexpression of enzymes and drug transporters capable of reducing the concentration of chemotherapeutics in the cytoplasm, allowing the cancer cells to repair damage caused by chemotherapy [48]. To solve this problem, it is necessary to explore and evaluate new molecules that are capable of eliminating cancer cells while having low levels of cytotoxicity against the cells of the healthy tissue surrounding the lesions. A promising possibility in this respect are bioactive cationic peptides (BCPs), which have emerged indirectly as an alternative for cancer treatment. BCPs are widely distributed in nature and are produced by almost all organisms as part of the nonspecific immune system [49–54]. These molecules were initially studied as potential substitutes for antibiotics. However, they have been shown to have a broad spectrum of target organisms ranging from viruses to parasites [55–58], and have the potential to treat polymicrobial biofilms [59,60]. BCPs are small molecules composed of up to 50 amino acids, making chemical synthesis and modification relatively easy. Moreover, although they vary significantly in structure and sequence, they share some general characteristics, being amphipathic and containing a high proportion of cationic and hydrophobic residues [56,61,62]. BCPs have been classified by their sequence and structure as either anionic or cationic, and rich in cysteine forming disulfide bonds, α-helices, β-sheets, cyclic, and linear (Figure 1) [63]. There is a wide diversity of BCPs, since their primary structures are very heterogeneous, leading to varied secondary structures. The vast majority of reported biologically active peptides are amphipathic and cationic at physiological pH, with charges from +3 to +9 [64,65].

**Figure 2:**
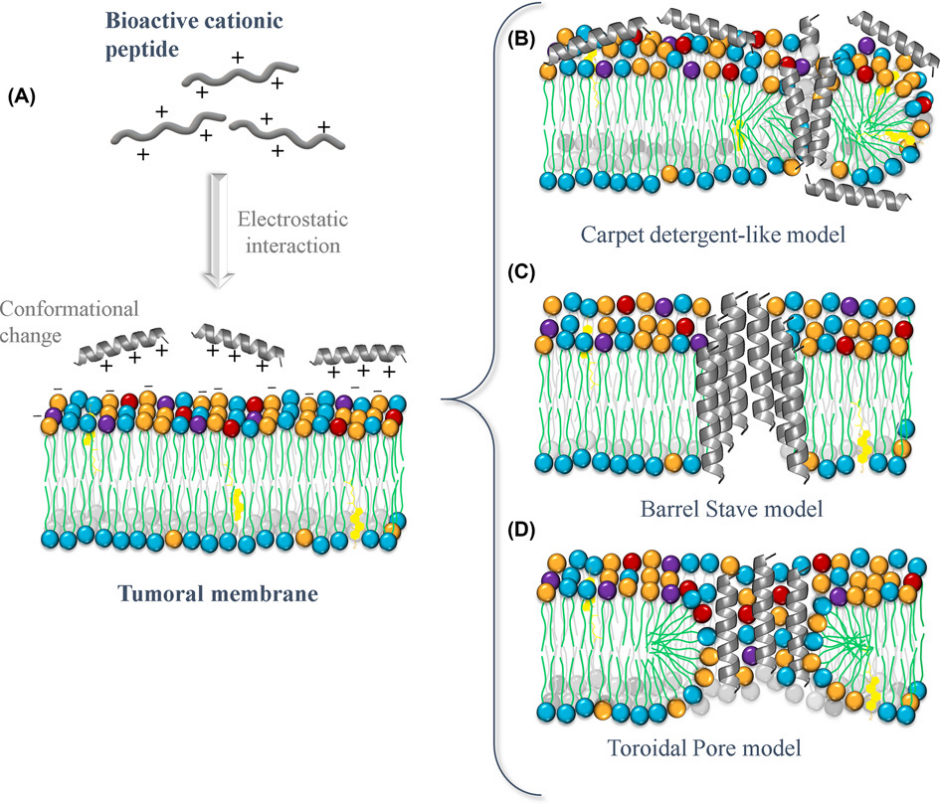
Schematic representation of the principal mechanism of action of BPCs Most peptides do not have a specific secondary structure in solution. Instead, the interaction with the membrane induces a conformational change in the peptide (**A**). After this electrostatic interaction, peptides disrupt the cell membrane through different modes of action. The most recognized modes are (**B**) carpet detergent-like model, (**C**) Barrel-stave, and (**D**) toroidal pore.

Different mechanisms of action have been proposed to explain how bioactive peptides exert their activity, all based on complex molecular interactions. However, the biological action of all of these mechanisms primarily involves altering the membrane of the target cells [66]. Therefore, peptides have become a promising potential agent in breast cancer treatment, since they reduce the generation of resistance mechanisms by cancer cells. Chemotherapeutics must enter cancer cells to exert their action, allowing the cells to develop resistance mechanisms to combat their effect. In contrast, one of the advantages of BCPs is that they act from outside the membrane, a mechanism that cannot be compensated for by tumor cells [67,68]. The mechanism of action of BCPs is composed of several stages, the first of which is mediated by electrostatic interactions between the positively charged residues of the peptide and the negatively charged groups of the tumor membrane [69]. After that, the hydrophobic interactions between the acyl chains of lipid membranes and non-polar residues then allow the incorporation of the peptide into the bilayer through various modes including the Barrel-stave, carpet detergent, and toroidal pore modes [63,70] (Figure 2). Although the later stages are based on the peptide’s ability to induce changes in the membrane, altering its structural properties and compromising its integrity, the first stage is considered fundamental in explaining the biological activity of the peptides and their potential selectivity [71]. Therefore, peptides induce instability and structural and physicochemical changes in the lipid bilayer, leading to cell death [72–74].

**Figure 3:**
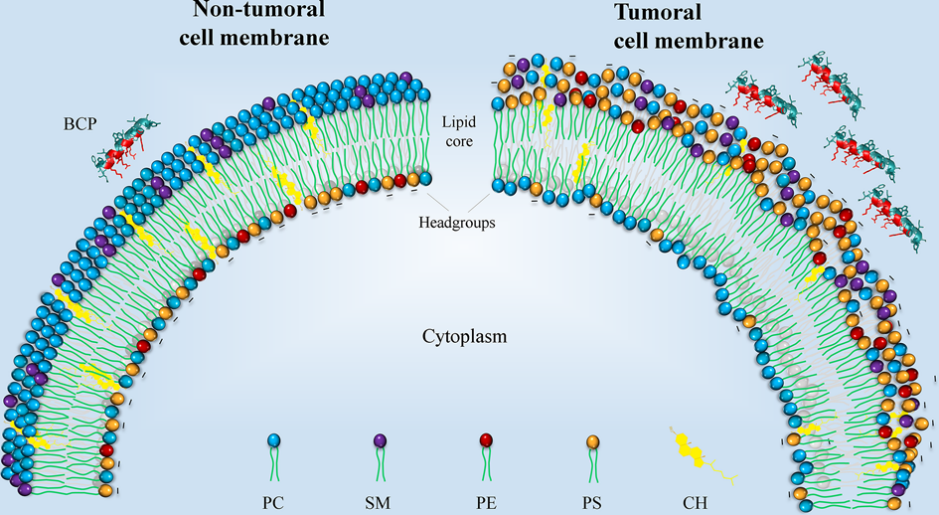
Schematic representation of non-tumoral and tumoral cell membranes The representation is based on the main differences in the outer membranes, including fluidity, cholesterol content, and lipid composition. Regarding the lipid composition, the non-tumoral membrane (left) is mainly composed of neutral lipids that do not interact with the BCPs. In contrast, the tumoral membranes (right) contain negatively charged lipids that interact with the positive residues of the BCPs, facilitating the recognition of cancer cells.

Furthermore, the higher phosphatidylserine (PS) concentration of cancer cell membranes favors electrostatic interaction between them and the peptides, unlike the membranes of normal cells that are considered neutral because they are mainly made up of zwitterionic lipids (Figure 3) [75,76]. Additionally, malignant cells are more fluid, and have lower cholesterol content tha the normal cells. Their lower cholesterol content makes malignant cells more susceptible to cell lysis by facilitating the destabilization of the membrane [69]. Leuschner et al. (2004) studied how the cholesterol content of eukaryotic cells acts as a protective factor against the cytolytic effect of BCPs [77,78]. Finally, several authors have reported that cancer cells present microvilli or cell membrane projections [79–81]. This would probably increase the surface area of cancer cells compared with normal cell membranes, which could in turn lead to increased interaction with BCPs [82]. However, this theory is still not proven. All these characteristics play a fundamental role in the selectivity of BCPs for malignant cells.

## Anticancer activities of BCPs

**Figure 4:**
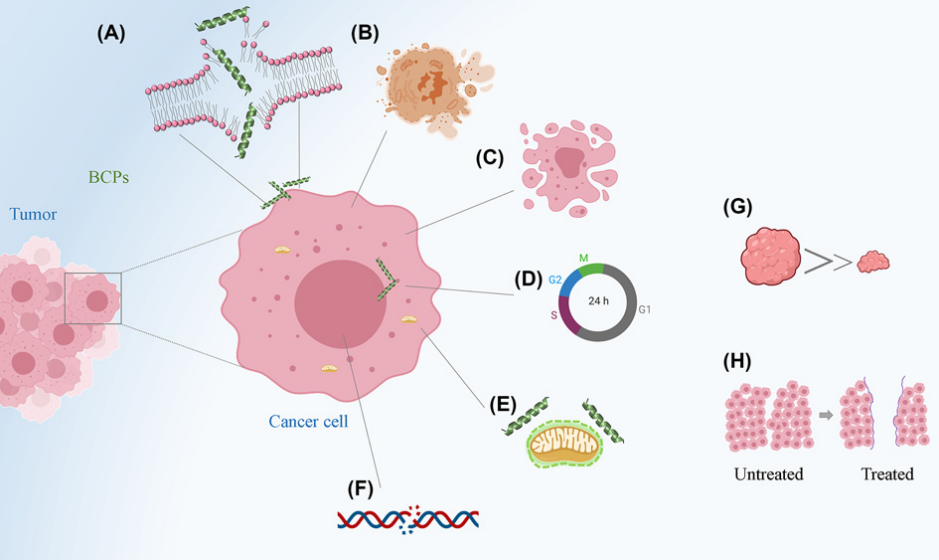
Schematic representation of BCP mechanism of action against breast cancer cells (**A**) Membrane disruption, (**B**) necrosis, (**C**) apoptosis, (**D**) cell cycle arrest, (**E**) mitochondria depolarization, (**F**) DNA fragmentation, (**G**) tumor growth reduction, and (**H**) inhibition of cell migration.

BCPs exhibit a wide range of anticancer activities. The main effects observed in various studies of the *in vitro* and *in vivo* models of breast cancer are cytotoxicity, antiproliferative activity, induction of cell death by necrosis or apoptosis, and inhibition of cell migration (Figure 4). The results of extensive research on the activity of cationic peptides against breast cancer are summarized in Table 1. An initial experimental approach model to evaluate the biological effects of BCPs against breast cancer includes *in vitro* cell-based analyses. Studies using cell line cultures have advantages, including easy maintenance, reproducibility of toxicity responses, and vast commercial availability of different cell types that allow the comparison of results between different treatment groups. Consequently, several breast cancer cell lines have been widely used for breast cancer modeling. Nevertheless, as shown in Table 1, MCF-7 and MDA-MB-231 cell lines are the most frequently employed in the associated studies [83].

**Table 1:** Experimental and epidemiological evidence of anticancer activities of BCPs against breast cancer

Peptide	Sequence	Charge	Dose	Experimental model	Main result	Reference
***In vitro***						
Bovine lactoferricin	FKCRRWQWRMKKLGAPSITCVRRAF	+8	0–100 μg/ml for 24 h	MDA-MB-231 cell line	Dose-dependent induction of DNA fragmentation indicative of apoptosis cell death	[84]
Bovine lactoferricin 6 (LfcinB6)	RRWQWR	+3	31 μM for 18 h	MDA-MB-231 cell line	Induction of cell death in 45% of population	[85]
pBmje	YNKKYRYHLKSCKKADK–NH_2_	+7	0–250 μM for 48 h	MCF-7 cell line	Dose-dependent cytotoxic activity with IC_50_ of 250 μM	[86]
Magainin II	GIGKFLHSAKKFFGKAFVGEIMNS	+3	0–120 μM for 72 h	MDA-MB-231 cell line	Dose-dependent cytotoxic activity with significant effect to 120 μM	[87]
Lysine-substituted VmCT1 analogs	FLGALWNVAKSVF–NH_2_ substitutions at positions 3, 7, and 11 in the hydrophilic face of VmCT1 amphipathic structure	From +2 (VmCT1) to +5	0.8–100 µM for 4 and 24 h	MCF-7 human breast cancer cells	Dose-dependent cytotoxic activity	[88]
IW13	IKHFKKQRRLIPW	+5	1, 3, 10, 30, 100 μM for 48 h	MCF-7 cell line	Cytotoxic assay showed EC_50_ values of 92 μM for MCF-7. The cationic antimicrobial peptide IW13 showed a high degree of selectivity compared with non-tumoral cells	[89]
Peptoid 1	H–(NLys-Nspe-Nspe)_4_–NH_2_	+4	0–50 μM for different time periods	MCF-7 cell line	Peptoid 1 exerted fast killing, the majority of cell death occurred within 4 h of treatment, and increased cytotoxicity was observed with longer treatments. IC_50_ for 72 h was 5 μM	[90]
Pseudhymenochirin-1Pa (Ps-1Pb) Pseudhymenochirin-2Pa (Ps-2Pa)	IKIPSFFRNILKKVGKEAVSLIAGALKQS GIFPIFAKLLGKVIKVASSLISKGRTE	+5 +4	1–100 µM for 24 h	MDA-MB-231 cell line	Ps-1Pb showed no selectivity for tumor cells, as the IC_50_ against non-neoplastic HUVEC cells (IC_50_: 5.6 µM) is in the same range as the values against MDA-MB-231 cells (IC_50_: 6.6 µM). In addition, the peptide is less cytotoxic to human erythrocytes than to the tumor cells. IPs-2Pa is strongly hemolytic against erythrocytes (IC_50_: 6 µM) but is appreciably less cytotoxic against HUVEC cells (IC_50_: 68 µM). It showed the same cytotoxic activity against MDA-MB-232 cells (IC_50_: 6.2 µM)	[91]
Amphipathic α-helical peptide	(KLAKLAK)_2_	+6	0–320 μM for 24 h	MCF-7, MDA-MB435S, MDA-MB453 cell lines	Dose–response cytotoxic effect for all tested cells. IC_50_ values were 88.1 μM for MCF7, 140 μM for MDA-MB435S, and 191 μM for MDA-MB453. Concerning PBL, non-tumoral cells, a selective effect was observed (IC_50_ > 320 μM)	[93]
Analogs of temporin-1CEa: LK1 LK2(5) LK2(6) LK3 LK2(6)A(L) LK2(6)AN(2L)	FVDLKKIANINSIKK–NH_2_ FKDLKKIANINSIKK–NH_2_ FVKLKKIANINSIKK–NH_2_ FKKLKKIANINSIKK–NH_2_ FVKLKKILNINSIKK–NH_2_ FVKLKKILNILSIKK–NH_2_	+4 +5 +6 +7 +6 +6	0–100 µM for 24 h	MCF-7, MDA-MB-231 and Bcap-37	LK2(6)A(L) and LK2(6)AN(2L) exhibited relatively stronger anticancer activities than temporin-1CEa and the other analogs. This may be due to their higher levels of both cationicity (+6) and hydrophobicity. These peptides reached the lowest IC_50_ for the three cell lines. The values were between 9 and 11 µM for MCF-7 and Bcap-37 and between 34 and 42 µM for MDA-MB-231	[94]
Kale (*Brassica alboglabra*) antifungal peptide	PEGPFQGPKATKPGDLAXQTWGGWXGQTPKY	+1	0–1.5 log concentrations for 72 h	MCF-7 cell line	Peptide inhibited the proliferation of MCF-7 cells with an IC_50_ of 3.4 µM	[103]
ERα17p	LMIKRSKKNSLALSL	+4	10 μM for 24 h	T47D, MDA-MB-231, MCF-7, and SK-BR-3 breast cancer cell lines	Proapoptotic effect. ERα-positive cells (MCF-7, T47D) were more sensitive to treatment than ER-negative cells (MDA-MB-231, SK-BR-3). The peptide decreased the number of colonies formed by cancer cells, indicative of an antiproliferative effect	[92]
Temporina-1CEa	FVDLKKIANIINSIF–NH_2_	+2	10–100 µM for 1, 6, 24 and 48 h	MCF-7 and MDB-MA-231 cell lines	Temporin-1CEa inhibited the proliferation of cancer cell lines in a dose-dependent manner. The IC_50_ values were 31.91 and 57.94 µM at 24 h for MCF-7 and MDA-MB-231 cells, respectively. Peptide caused a concentration-dependent increase in the release of LDH in MCF-7 cells. TEM studies showed disrupted membrane, and condensed and hollow nuclei, which caused leakage of the intracellular contents	[95]
CDAK	DGRCLLIIKLAKLAKKLAKLAK	+6	10–200 µg/ml for 24, 48, and 72 h	MCF-7 and MDB-MA-231 cell lines	Dose-dependent cytotoxicity effect in MCF-7 (190 µg/ml and MDA-MB-231(212 µg/ml) cells. Peptide treatment increased the percentage of apoptotic cells in both cell lines and the formation of DNA nucleosome ladders in both cell lines was detected. Caspase-3 was 8.5 and 2.8 higher, and Bcl-2 96 and 92% lower, respectively, in MCF-7 and MDA-MB-231 cells treated with CDAK, compared with control	[96]
pHLIP-(KLAKLAK)_2_ construct	KLAKLAKKLAKLAK	+6	From 10 µM down to 2.5 nM at either pH 7.4 or 5.0 for 2 h	MDB-MA-231	pHLIP-(KLAKLAK)_2_ was cytotoxic against MDB-MA-231 cell with an IC_50_ value of 1 µM. This peptide does not cause cell death through dramatic disruption of the plasma membrane, but a lower pH disrupts the plasma membrane and disrupts the mitochondrial membrane	[98]
Maculatin 1.1 (Mac1)	GLFGVLAKVAAHVVPAIAEHF–NH_2_	+1	0.35–40 µM for 2h	MCF-7 cell line	An IC_50_ value of 23 µM. Membrane disruption is the probable mode of action	[99]
NS	PKKKRKVWKLLQQFFGLM–NH_2_	+7	0–20 µM for 24 h	MDA-MB-231 cell line	NS could kill tumoral cells in a dose-dependent manner (IC_50_: 10 µM) and exhibited a cytotoxic effect via membrane disruption	[100]
EVP50	RhoB–KRFKKFFKK	+6	0–40 μM for 1 h	MCF-7 and MDA-MB-431 cell lines	Treatment significantly decreased the viability and increased the cytotoxicity of cells in a dose-dependent manner. Treatment of MCF-7 cells for 5 min compromised the cell membrane and caused cytosolic calcium to increase	[101]
NRC-03 NRC-07	GRRKRKWLRRIGKGVKIIGGAALDHL–NH_2_ RWGKWFKKATHVGKHVGKAALTAYL–NH_2_	+9 +7	5–50 µM for 24 h	MDA-MB-231, MDA-MB-468, T47-D, SKBR3, MCF-7 and paclitaxel-resistant MCF-7 (MCF-7-TX400) breast cancer cells	SKBR3, MDA-MB-468, and 4T1 cells were more susceptible to NCR-03 and NCR-07 than T47-D, MDA-MB-231, and MCF-7 cells, which required 2.5–10-times more NCR-03 and NRC-07 to cause significant cytotoxicity. NRC-03 or NRC-07 killed primary cultures of human dermal fibroblasts or HUVECs, and did not exhibit hemolytic activity. Peptides induced cell death by a membranolytic mechanism and pore formation in mitochondria	[102]
TP4	H-FIHHIIGGLFSAGKAIHRLIRRRRR–OH	+7	2.5–20 µg/ml at different time points, 3, 6, 12 and 24 h	MDB-MA-231, MDB.MA-453 and MCF-7 cell line	Treatments with 15 µg/ml (5.03 µM) of TP4 are sufficient to kill over 50% of breast cancer cells at 6 h. Lactate dehydrogenase (LDH) increased at 3 h post-TP4 treatment in TNBC cells, indicating that peptide induces necrotic death in TNBC cells. TP4 binds to the mitochondria, disrupts Ca^2+^ homeostasis, and ultimately induces FOSB protein	[104]
Vitamin E succinate modified octaarginine-octahistidine (VES-H8R8)	VES–HHHHHHHHRRRRRRRR	+8	5, 10, and 20 μM for different times	EMT6/P and EMT6/AR-1 (doxorubicin-resistant) breast cancer cells	Selective activity with IC_50_ on EMT6/P of 4.4 μM, and IC_50_ on EMT6/AR-1 of 7.3 μM, compared with NIH/3T3 non-tumoral cells, with IC_50_ close to 40 μM. Cytotoxic to cancer cells by mitochondria depolarization, increased ROS production, reduced cell bioenergetics, triggering apoptosis, and G_1_ cell cycle arrest	[105]
Temporin-1CEa	FVDLKKIANIINSIFGK	+3	20–40 µM for 1 h	Bcap-37 human breast cancer cell line	Rapid cell death in a concentration-dependent manner. Cell death mechanisms were associated with rapid intracellular Ca^2+^ leakage, the collapse of mitochondrial membrane potential, and overgeneration of ROS	[106]
Aurein 1.2	GLFDIIKKIAESF–NH_2_	+1	0–32 μM for 12 or 24 h	MCF-7 cells and MX-1 cell lines	The IC_50_ value was less than 8 μM in MCF-7 cells and less than 20 μM in MX-1 cells. Peptide exhibited relatively higher cytotoxicity against breast cancer cells than against normal cells (IC_50_ > 60 μM). Significant apoptotic activity was detected by annexin V-FITC/PI staining	[107]
Buforin IIb	RAGLQFPVGRLLRRLLRRLLR	+7	0–32 μM for 12 or 24 h	MCF-7 cells and MX-1 cell lines	The IC_50_ value was less than 8 μM in MCF-7 cells and less than 20 μM in MX-1 cells. Peptide exhibited relatively higher cytotoxicity against breast cancer cells than against normal cells (IC_50_ > 60 μM). In MCF-7 cells, significant apoptotic activity was detected by annexin V translocation, DAPI staining, and the activation of caspase-9 and cleavage of PARP	
BMAP-28m	GGLRSLGRKILRAWKKYGIPIVPIIRI–NH_2_	+7	4–60 µM for 24 h	MCF-7 and MX-1 cell lines	Dose-dependent cytotoxicity IC_50_ less than 8 µM in MCF-7 cells and less than 20 µM in MX-1 cells. Treatments induce PS exposure, which was related to the apoptotic activity	
Chimeric protein p28-NRC	LSTAADMQGVVTDGMASS GLDKDYLKPDDPAPAPAAPAPAPLHDL AAGGIIKVGKGIRRLWKRKRRG	+4	0.5–8 µM for 48 h	MCF-7 and MDA-MB-231 cell lines	p28-NRC killed MCF-7 and MDA-MB-231 in a dose-dependent manner, with IC_50_ values of 1.88 and 1.89 µM, respectively. Increased expression levels of proapoptotic genes *AIF, BAX*, and *Caspase-3*, and decreased anti-apoptotic gene *Bcl-2*	[108]
[G10a]SHa-BCTP conjugate	FLSGIVGML–D–Ala–KLF–NH_2_–WLEAAYQKFL	+1	25, 50, and 100 µM for 48 h	MCF-7 human breast cell line	[G10a] SHa-BCTP conjugate was active against the MCF-7 cell line (IC_50_: 26.85 µM) without cytotoxicity against non-cancerous cells (IC_50_ > 100 µM). Treatments induced high fragmentation of DNA and triggered apoptotic cell death in a dose-dependent manner. Down-regulating expression of Bcl-2 and up-regulating BAX and caspase-3 were observed	[109]
Melittin	GIGAVLKVLTTGLPALISWIKRKRQQ	+5	0–10 μM for 24 h	Panel of human and murine breast cancer cell lines	Melittin was significantly more potent against HER2-enriched breast cancer and TNBC compared with normal cells. Cytotoxic effect was related to the suppression of activation of EGFR and HER2 by interfering with the phosphorylation of these receptors in the plasma membrane of breast carcinoma cells	[97]
			0–20 μg/ml for 24 and 48 h	MDA-MB-231 cell line	Dose-dependent cytotoxic activity with IC_50_ of 15 μg/ml. Reduced DNA synthesis at S phase and increased G_1_/S transition, with related low expression of mRNA and protein level of Cyclin D1. Time-dependent alterations in the chromatin morphology of the treated cells, which are related to apoptosis. Co-delivery of melittin with miR-34a increased cell death induction	[110]
LTX-315	KKWWKKWDipK–NH_2_	+6	0–20 μg/ml for 24 and 48 h	MDA-MB-231 cell line	Dose-dependent cytotoxic activity with IC_50_ of 150 μg/ml. Reduced DNA synthesis at the S phase and increased G_1_/S transition. Time-dependent alterations in the chromatin morphology of the treated cells, which are related to apoptosis	
FR8P FR11P	FRRFFKWPRRFFKFF–NH_2_ FRRFFKWFRRPFKFF–NH_2_	+6 +6	0–70 µM for 24 h	MDA-MB-231 cell line	Depolarized the mitochondrial transmembrane potential in a dose-dependent manner, indicative of induction of intrinsic pathway of apoptosis. Both peptides induced G_2_/M phase cell arrest in a concentration-dependent manner. Down-regulation of P44/42 protein MAP kinase proteins responsible for the migration of breast cancer cells	[111]
PR39	RRRPR PPYLPRPRPPPFFPP RLPPRIPPGFPPRFPPRFP–NH_2_	+11	9 and 18 μM for 48 h	4T1 cells (Stat3 knockdown)	Treatment significantly inhibited 4T1 cell invasion and migration, and it was estimated that PR39 and Stat3 siRNA could have a synergistic effect on the invasion and migration of 4T1 cells	[112]
MAP-04-03	KWLRRVWRWWR–NH_2_	+6	25, 50, 75, and 100 µM for 24 and 48 h	MCF-7 cell line	The IC_50_ value was 61.5 µM in the cell viability assay. Effectively inhibited cell migration at 5 µM, which indicates potency ten times that of IC_50_	[113]
***In vivo***						
Peptoide 1	H–(NLys–Nspe–Nspe)_4_–NH_2_	+4	1 mg/kg three-times per week	NSG mice with an orthotopic injection of cells from a dissociated second-generation metastatic breast cancer tumor	Peptoid 1 significantly inhibited tumor growth. Furthermore, the applied dosages of peptoids did not cause any noticeable acute toxicity in mice	[90]
Melittin	GIGAVLKVLTTGLPALISWIKRKRQQ	+5	5 mg/kg, treatment every 2 days from day 3, with seven treatments in total	BALB/c mice with an injection of murine p53- TNBC cell line T11	Melittin reduces tumor volume. In combination with docetaxel treatment, tumor control was enhanced	[97]
Amphipathic α-helical peptide	(KLAKLAK)_2_	+6	250 μg in 50 μl PBS weekly	MDA-MB435S breast cancer-bearing nude mice	Peptide treatment inhibits tumor growth and prolongs overall survival	[93]
ERα17p	LMIKRSKKNSLALSL	+4	50 mM or 1.5 mg/kg diluted in PBS, three times per week	Male BalbC^−/−^ nude mice injected with MDA-MB-231 cells	After 4 weeks of treatment, a reduction in tumor size of more than 50% was observed after ERα17p treatment when compared with untreated tumors. The histological analysis of the tumors revealed a massive ERa17p-induced central necrosis	[92]
TP4	H–FIHHIIGGLFSAGKAIHRLIRRRRR–OH	+7	A group of nude mice with xenografts were treated with TP4 (500 µg in 50 µl distilled water plus 10 µl KY jelly) 14-times every two days once the tumor reached a specific size	TNBC cells were subcutaneously transplanted into nude mice (*n*=5) and assessed tumor growth daily for 28 years	Intratumoral injection of TP4 caused extensive necrosis of TNBC in xenograft tumors without causing adverse side effects. FOSB expression was also detected within the tumor	[104]
NRC-03 NRC-07	GRRKRKWLRRIGKGVKIIGGAALDHL–NH_2_ RWGKWFKKATHVGKHVGKAALTAYL–NH_2_	+9 +7	When the tumors reached a volume greater than 120 mm^3^, mice were administered 20 µl of the HBSS vehicle or 0.5 mg NCR-03 or NRC-07 in 20 µl of HBSS by intratumoral injection on days 1, 3, and 5	NOD SCID mice were engrafted with MDA-MB-231 cells by subcutaneous injection in one hind flank	Treated tumors were significantly smaller than control tumors at day 12. Histologic analysis revealed that the necrotic core of peptide-treated tumors was more significant than that of control tumors. Intertumoral delivery of NRC-03 and NRC-07 to mice did not have any noticeable adverse side effects	[114]
Buforin IIb	RAGLQFPVGRLLRRLLRRLLR	+7	2.5 and 5 mg/kg. Peptide was injected through the tail vein of mice on days 1, 4, 8, and 12	BALB/c nude mice injected with MX-1 cells	Treatment significantly suppressed the growth of xenograft tumors. H&E staining showed nuclear shrinkage in the treatment group. In addition, cells from tumors treated stained positive for TUNEL. Fewer CD31^+^ cells were detected in tumors treated with 5 mg/kg buforin IIb, which is associated with inhibition of vascularization	[107]
CDAK	DGRCLLIIKLAKLAKKLAKLAK	+6	When the tumor reached 60 mm^3^ in size, the mice were randomized into three groups: (1) CDAK (4 mg/kg); (2) CRLK (4 mg/kg); and (3) saline (control). They were then injected intravenously (50 ml/injection) three times a week for 3 weeks	MDA-MB-231 cells were injected subcutaneously into the right flank of 6- to 9-week-old female BALB/cnu-nu athymic nude mice.	The tumors treated with CDAK were significantly smaller than the control group. CDAK significantly inhibited tumor angiogenesis.	
LTX-315	KKWWKKWDipK–NH_2_	+6	0.5–1.0 mg peptide/50 μl saline once a day for 2– 3 consecutive days	Balb/C wild-type mice with orthotopic injection of 4T1 cells in mammary fat pad.	Co-treatment with doxorubicin induced strong local necrosis and immune-mediated changes	[115]
**Epidemiological**						
LTX-315	KKWWKKWDipK–NH_2_	+6	2–7 mg per lesion injection. LTX-315 was administered on days 1, 2, and 3 during the first week and subsquently once weekly for a total of 6 weeks. The maintenance phase included one injection per day every 2 weeks for 20 weeks	Phase I trial in patients with breast cancer (*n*=8)	Intratumoral injection of LTX-315 was tolerated well. However, the dosing regimen of LTX-315 induced necrosis and CD8^+^ T-cell infiltration into the tumor microenvironment	[116]

Cytotoxic effect on MCF-7 or MDA-MB-231 cancer cell lines has been reported for the cationic peptides Bovine lactoferricin [84], its Bovine variant lactoferricin 6 [85], pBmje [86], Magainin II [87], the Lysine-substituted VmCT1 analogs [88], IW13 [89], Peptoide 1 [90], Pseudhymenochirin-1Pa and Pseudhymenochirin-2Pa [91]. Moreover, cell proliferation assays revealed that Kale antifungal peptide impaired the proliferation of MCF-7 cells. In addition, ERα17p peptide decreased the number of colonies formed by different cancer cells, indicative of an antiproliferative effect [92]. However, the authors of these studies concluded that the peptides had a dose-dependent cytotoxic or antiproliferative activity without thoroughly investigating the mechanism of death induction.

Other researchers evaluated the differential response to BCPs in MCF-7 and MDA-MB-231 cell lines due to their important phenotypic variations. MCF-7 is estrogen receptor-positive (ER+) and progesterone receptor-positive (PR+). On the other hand, MDA-MB-231 is estrogen receptor-negative (ER−) and progesterone receptor-negative (PR−). In general, treatments with BCPs significantly decreased the viability of both types of cells in a dose-dependent manner, and, as is evident in IC_50_ values, receptor-positive MCF-7 cells were more sensitive to peptide treatments than receptor-negative cells (MDA-MB-231) [92–96]. Additional evidence reported by Duffy and Sorolla [97] showed that melittin was significantly more potent against HER2-enriched breast cancer cells. Cytotoxic effect was related to the suppression of activation of EGFR and HER2 by interfering with the phosphorylation of these receptors in the plasma membrane of breast carcinoma cells [97].

As described previously in this review, the mechanism of action of BCPs in targeting cell membranes is based on electrostatic interactions between the cationic residues on the peptide and anionic lipids on cancer cell membranes. In this respect, several authors have suggested that the mode of action is probably dependent on membrane disruption and subsequent induction of necrosis, as was reported for breast cancer cells treated with Temporina-1CEa [95], pHLIP-(KLAKLAK)_2_ construct [98], Maculatin 1.1 [99], NC peptide [100], EVP50 [101], and NRC-03 NRC-07 peptides [102].

After the action of BCPs on the cell membrane, the peptides can also infiltrate intracellular spaces. Hence, the biological effects of BCPs are also associated with the targeting of other cellular structures, such as mitochondria [117–119], as well as interference with signaling pathways linked to apoptosis cell death [66] and cell cycle [120,121]. Many BCPs are reported to induce these cellular changes. For example, Ting et al*.* reported that, in MDB-MA-231 cells treated with TP4 peptide, while the induction of DNA fragmentation or caspase 3 activation after treatment was not evident, lactate dehydrogenase (LDH) increased at 3 h post-TP4 treatment in TNBC cells, indicating that this peptide induces necrotic death in TNBC cells. Furthermore, the mechanism action of TP4 showed that it binds to the mitochondria, disrupts Ca^2+^ homeostasis, and ultimately induces FOSB protein to activate TNBC cell death [104]. Another study reported that VES-H8R8 peptide is cytotoxic to breast cancer cells through mitochondria depolarization, increased reactive oxygen species (ROS) production, reduced cell bioenergetics, and triggering of apoptosis G_1_ cell cycle arrest [105]. Similarly, Wang et al. observed that Temporin-1CEa induces cell death, which is associated with rapid intracellular Ca^2+^ leakage, collapse of mitochondrial membrane potential, and overgeneration of ROS [106]. Figure 4 summarizes all the proposed mechanisms for the BCPs.

Aurein 1.2, Buforin IIb, and BMAP-28m induce apoptotic cell death, as was evidenced in MCF-7 cells, where peptides provoked PS exposure in treated cells. Additionally, Bufforin IIb activity was associated with activation of caspase-9 and cleavage of PARP [107]. Soleimani et al. reported that chimeric protein p28-NRC induces cell injury in MCF-7 and MDA-MB-231 in a dose-dependent manner, with increased expression levels of the proapoptotic genes *AIF*, *BAX*, and *Caspase-3*, and decreased expression of the anti-apoptotic gene *Bcl-2* [108]. Similar results were published previously for [G10a]SHa–BCTP conjugate peptide, where treatment induced high DNA fragmentation, down-regulating the expression of Bcl-2, and up-regulating BAX and caspase-3 [109].

Many chemotherapeutics affect cancer cells by altering the cell cycle, generally in specific control points; indeed, some BCPs have been reported to affect the growth and division of breast cancer cells. For instance, in MDA-MB-231 cells, melitinin reduced DNA synthesis at the S phase and increased G_1_/S transition, with related low expression of mRNA and protein level of the regulator protein Cyclin D1. Similarly, LTX-315 showed increased G_1_/S transition and time-dependent alterations in the chromatin morphology of the treated cells, which is related to apoptosis [110]. FR8P and FR11P peptides induced G_2_/M phase cell arrest in MDA-MB-231 cells, linked to depolarization of mitochondrial membrane potential and activation of caspases [111].

Since metastasis is responsible for therapeutic failure, molecules that can specifically interfere in the cell migration process are helpful for cancer treatment. Various BCPs with capacity to inhibit cell migration in breast cancer cells have been reported. For example, PR39 treatment significantly inhibited 4T1 cell invasion and migration, and it was suggested that it could have a synergistic effect with Stat3 siRNA, efficiently inhibiting cellular proliferation and migration [112]. FR8P and FR11P peptides also induce a down-regulation of the P44/42 MAP kinase protein responsible for the migration of breast cancer cells [111]. Another study reported an IC_50_ value of 61.5 µM for MAP-04-03, although the peptide was very effective at inhibiting the cell migration at 5 µM, with inhabitation of approx. 40% of cell migration [113].

The biological effects induced by BCPs also have been evaluated *in vivo* controlled environments using animal testing. Rats and mice injected with breast cancer cells are the most common model for tumors. *In vivo* models employing BCP treatments significantly inhibited tumor growth, as was reported for peptoid 1 [90], melittin [97], and amphipathic α-helical peptide [93]. In other reports, tumor growth reduction was linked to necrosis, for example in ERα17p [92], TP4 [104], and NRC-03 and NRC-07 peptides [102]. Further, vascularization and angiogenesis inhibition in xenograft tumors were reported after buforin IIb [107] and CDAK [96]. The co-treatment of BCPs with standard chemotherapeutics also have been evaluated. In breast cancer, LTX-315 in co-treatment with doxorubicin induced substantial local necrosis and immune-mediated changes in the tumor microenvironment, followed by complete regression in most animals treated [122]. Encouragingly, most of the *in vivo* studies found that BCP treatment did not have any noticeable adverse side effects. Despite several studies on the discovery or design of anticancer peptides against breast cancer, only LTX-315 is tested in clinical trials. Results of Phase I trial in eight patients with breast cancer (NCT01986426) show that intratumoral injection of LTX-315 is well tolerated. The dosing regimen used for LTX-315 induces necrosis and CD8^+^ T-cell infiltration into the tumor microenvironment [116].

## Current status and future directions

Breast cancer continues to be one of the leading causes of women’s deaths worldwide. The search for new therapies for this disease is a priority, especially in view of the very well-known side effects of traditional treatments. Although researchers have been studying the potential of BCPs for cancer treatment, there are still some critical barriers to overcome. Firstly, the selectivity of most BCPs is not sufficiently differentiated between cancer cells and normal cells, resulting in limited clinical applications. Second, the low resistance of BCPs to proteolytic cleavage is one of the aspects of peptides that has raises the most questions. It explains their short half-life and, therefore, low bioavailability *in vivo* [123], a limitation that avoid using peptides as pharmaceutical agents.

However, different pharmaceutical companies have made progress in evaluating and developing drugs from natural or modified peptides, demonstrating the potential use of these compounds. This potential is based on the easy modification of the sequence, net charge, hydrophobicity, amphipathicity, and therefore the peptide’s secondary structure. Some of the more unique peptides have reached phase II and III clinical studies, and are intended for use topically or intravenously to treat localized and systemic infections [124]. This is the case with the peptide derived from lactoferrin hLF-1-11 (AM-Pharma), for use in the treatment of transplant-associated infections; the peptide PAC113, based on histatin 5 (PacGen) from human saliva and used for the treatment of oral candidiasis; and the peptide Mersacidin (Novacta Biosystems Ltd), derived from bacteriocin and used for the treatment of infections of Gram-positive bacteria [124]. One of the most promising peptides developed in recent years is the synthetic peptide LTX-315, a derivative of lactoferricin, known by its trade name as Oncopore™, which is active in several cancer cell lines and is in phase II clinical trials [125]. LTX-315 lyses cancer cells (necrosis) through a membrane destabilizing mechanism followed by the release of danger-associated molecular patterns (DAMPs), thereby reprogramming the tumor microenvironment while presenting low cytotoxicity against human erythrocytes [118,126]. The results using a fibrosarcoma model have shown that 80% of animals treated with LTX-315 show regression in the size of the treated tumor [115,127]. Currently, it is considered an alternative treatment for different types of cancer, but it is mainly used in melanoma. The development of this peptide was the basis for the foundation of the company Lytix Biopharma, whose objective is the pharmacological development of oncolytic peptides [128]. The next generation of peptides will be based on modifications focused on improving the cancer targeting, specificity, and efficacy of peptides, reducing their potential side effects.

## Abbreviations

BCP: bioactive cationic peptide
HER2: human epidermal growth factor receptor 2
IARC: International Agency for Research on Cancer
PS: phosphatidylserine
ROS: reactive oxygen species
TNBC: triple-negative breast cancer
WHO: World Health Organization
